# Explaining patient delay in healthcare seeking and loss to diagnostic follow-up among patients with presumptive tuberculosis in Tanzania: a mixed-methods study

**DOI:** 10.1186/s12913-019-4030-4

**Published:** 2019-04-05

**Authors:** Grace Mhalu, Mitchell G. Weiss, Jerry Hella, Francis Mhimbira, Enos Mahongo, Christian Schindler, Klaus Reither, Lukas Fenner, Elisabeth Zemp, Sonja Merten

**Affiliations:** 1 Ifakara Health Institute, Dar es Salaam and Bagamoyo, Tanzania; 2 Swiss Tropical and Public Health Institute, Basel, Switzerland; 3 University of Basel, Basel, Switzerland; 4 Institute of Social and Preventive Medicine, University of Bern, Bern, Switzerland

**Keywords:** Tuberculosis, Patient delay, Loss to follow-up, Help-seeking behaviour, Gender, Cultural epidemiology

## Abstract

**Background:**

Delay in healthcare seeking and loss to diagnostic follow-up (LDFU) contribute to substantial increase in tuberculosis (TB) morbidity and mortality. We examined factors, including perceived causes and prior help seeking, contributing to delay and LDFU during referral to a TB clinic among patients with presumptive TB initially seeking help at the pharmacies in Dar es Salaam Tanzania.

**Methods:**

In a TB clinic, a semi-structured interview based on the explanatory model interview catalogue (EMIC) framework for cultural epidemiology was administered to presumptive TB patients enrolled at pharmacies during an intervention study. We assessed delay in seeking care at any medical care provider for a period of ≥3 weeks after the onset of symptoms, LDFU during referral (not reaching the TB clinic), and LDFU for three required TB clinic visits among the presumptive and confirmed TB patients. Logistic regression models were used to assess factors associated with delay and LDFU.

**Results:**

Among 136 interviewed patients, 86 (63.2%) were LDFU from pharmacies and TB clinic while 50 (36.8%) were non-LDFU. Out of 136 patients 88 (64.7%) delayed seeking care, of whom 59 (67%) were females. Among the 86 (63.2%) patients in LDFU group, 62 (72.1%) delayed seeking care, while among the 50 (36.8%) non-LDFU, 26 (52.0%) had also delayed seeking care. Prior consultation with a traditional healer (aOR 2.84, 95% CI 1.08–7.40), perceived causes as ingestion (water and food) (aOR 0.38 CI 0.16–0.89), and substance use (smoking and alcohol) (aOR 1.45 CI 0.98–2.14) were all associated with patient delay. Female gender was associated with LDFU (aOR 3.80, 95% CI 1.62–8.87) but not with delay. Other conditions as prior illness and heredity were also associated with LDFU but not delay (aOR 1.48 CI 1.01–2.17).

**Conclusion:**

Delay and LDFU after referral from the pharmacies were substantial. Notable effects of diagnosis and female gender indicate a need for more attention to women’s health to promote timely and sustained TB treatment. Public awareness to counter misconceptions about the causes of TB is needed.

## Background

Tuberculosis (TB) ranks above HIV/AIDS as the number one cause of death from infectious-disease worldwide accounting for 1.7 million TB deaths and 10.4 million new cases in 2016 [1]. Tanzania is among the 30 countries with a high TB burden where TB case detection relies on passive case findings. Although Individuals with TB symptoms may present themselves to healthcare facilities for TB diagnosis [2–4] the problem of delayed appropriate help seeking for people with TB symptoms is widespread in sub-Saharan African countries [5]. The problem of diagnosis and starting appropriate treatment is further complicated by early loss to diagnostic follow up [6]. In view of good prospects for cure with timely diagnosis and treatment, these are serious problems [2, 6].

Diagnosis and treatment of TB patients require recognition of symptoms and both prompt presentation for diagnosis and treatment in a competent healthcare facility. Furthermore, healthcare must be sustained to limit morbidity and mortality [7, 8]. People with symptoms of TB usually follow a complex pathway to care, typically including prior consultation with two or three healthcare providers before reaching specialized healthcare facilities capable of diagnosing and treating TB [9–11]. Research suggests that factors contributing to delay in reaching specialized healthcare facilities and loss to diagnostic follow-up (LDFU) include dissatisfaction with health services, use of self-prescribed medication and initial help seeking with traditional healers [12–14]. LDFU leads to poor health outcomes for the individuals such as increased morbidity, decreasing the quality of life and increasing mortality [15]. It also enables further spread of TB.

Most studies addressing loss to follow-up (LTFU) consider TB patients in treatment, but little is known about LDFU for the larger group of people with presumptive TB symptoms who have not yet been diagnosed and not yet started treatment [15, 16]. To explain delayed healthcare seeking and LDFU that complicates diagnosis and initiating TB treatment, we examined the role of selected features of patients’ own illness explanatory models, including patient-perceived TB causes and prior healthcare seeking [17]. We interviewed people with symptoms of TB who were referred for diagnosis but did not come for the three recommended follow-up visits and assessed patient delay in seeking care, and we compared them with TB patients who completed recommended diagnostic follow-up in a TB referral clinic in Dar es Salaam Tanzania, to identify factors associated with delay and LDFU.

## Methods

### Study setting

#### Intervention study: tuberculosis case findings at the pharmacies

**Fig. 1:**
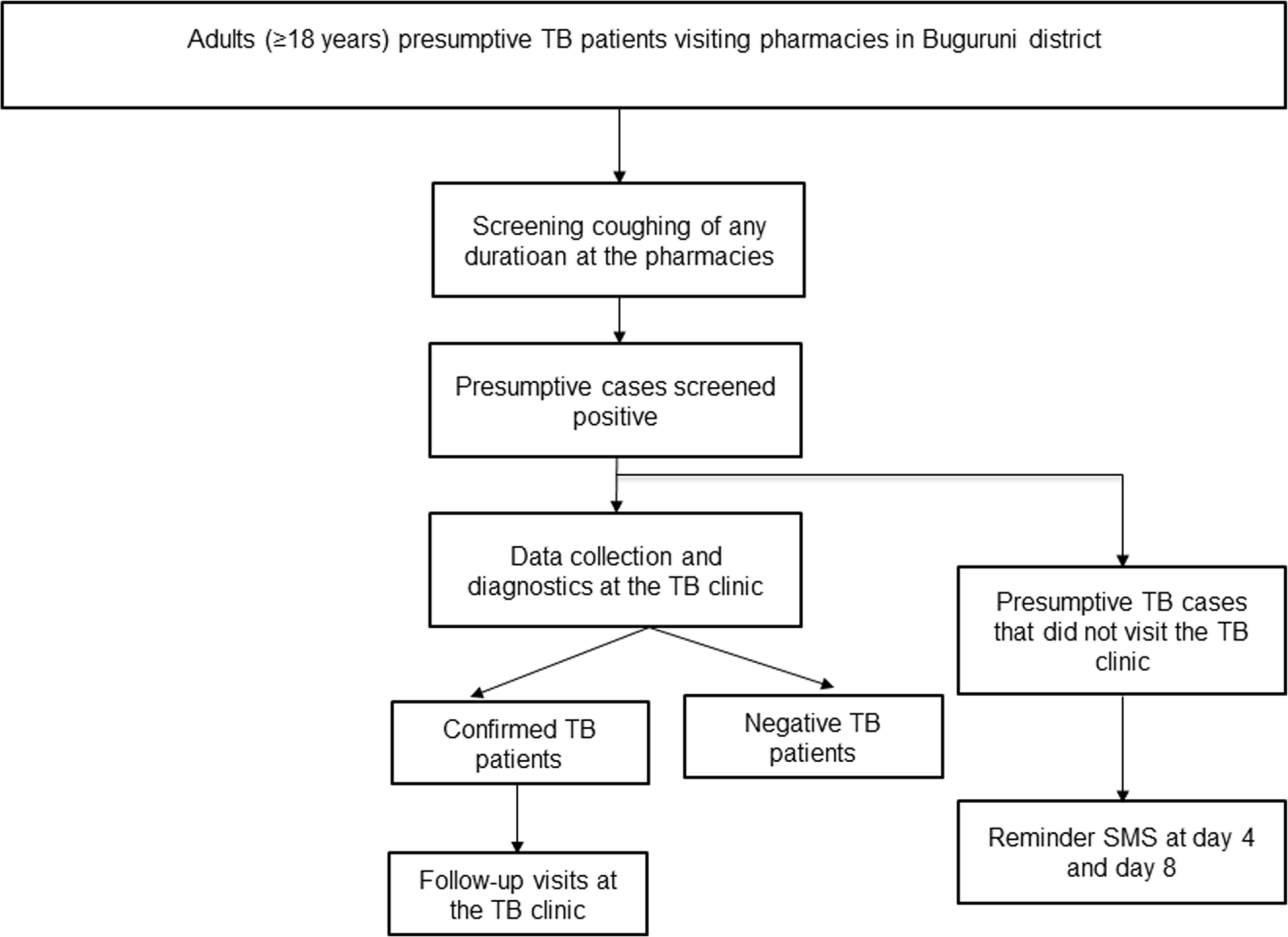
Intervention study (TB-PHARM) patient referral flowchart

A study of TB case finding at pharmacies (TB-PHARM) was conducted in 2015 in the Ilala district of Dar es Salaam, Tanzania. Trained pharmacists who used an electronically monitored referral system participated in the study [18]. The study aimed at developing and evaluating referrals of presumptive TB patients from pharmacies to TB clinics of the National Tuberculosis and Leprosy Programme (NTLP). Selection of the pharmacies was based on the geographical location to the TB clinic, the ownership (government, private) and the size of the pharmacy to obtain a representative mix. The intervention study included all pharmacy clients aged 18 years or older visiting selected pharmacies in the Ilala district with TB symptoms (coughing of any duration). Pharmacy clients who bought antibiotics for patients with cough at home were encouraged to bring their patients so they can be reffered to the TB clinic Identified presumptive TB patients were counseled and referred to a TB diagnostic centre with a referral card. The patient flow of the intervention study is schematically shown in Fig. 1. Individuals who were currently investigated for TB, as well as TB patients on treatment or who had completed treatment within the last 3 months were excluded from the intervention study.

#### Short overview of LDFU sub-study

The LDFU sub-study included a fraction of the presumptive and confirmed patients that participated in the big TB-PHARM study. This was an exploratory study that aimed at exploring factors associated with patient delay and loss to diagnostic follow-up in the intervention study (TB-PHARM). We employed an integrated approach to a mixed-methods study design, which is a characteristic feature of cultural epidemiological studies that integrate acquisition and analysis of quantitative and qualitative data [19–21].

#### LDFU-study setting

The LDFU sub-study was conducted in Ilala district (Buguruni hospital) in Dar es Salaam Tanzania. Ilala has a population of 1,220,611 with an average household size of 4.0 persons (Tanzania census of 2012). The district notified 4582 TB cases (all forms) in 2012. The study setting was a residential area in Buguruni sub-district with a population of 70,585 and 559 notified TB cases in 2012. There is one registered TB diagnostic and treatment centre in the study area.

#### LDFU-study population

We established a patient tracing system that included community health workers, Ex-TB patient’s organisations and clinical workers. We traced all presumptive and confirmed TB patients who did not visit the TB clinic after referral from the pharmacies or those who did not complete their required three visits at the clinic after treatment initiation. The list of all LDFU was obtained from the main database of the intervention study. We further included confirmed TB patients from the main database of the intervention study who were not LDFU and completed all the required three visits at the TB clinic. Patient delay was assessed in all the LDFU and non-LDFU patients and was defined as delay to visit a healthcare provider including pharmacies, hospitals, dispensaries and primary healthcare centers of 3 weeks or more after the onset of symptoms.

### Instrument

In a mixed-methods approach, we used a semi-structured explanatory model interview based on the framework of the explanatory model interview catalogue (EMIC) for cultural epidemiology to study patients’ illness explanatory models [22]. The interview and coding categories were adopted from an earlier EMIC interview developed and used in a WHO/TDR-supported multi-country study of gender and tuberculosis in India, Bangladesh, Malawi and Colombia [23]. The various sections of the interview inquired about priority symptoms, perceived causes (PCs) and place and timing of help seeking, acknowledging delay. Open questions were asked first, and responses were coded according to coding options. Coding distinguished categories reported in spontaneous responses to open questions from categories reported only in response to probed categories, which had not been mentioned spontaneously. Narrative elaboration was included as a qualitative component of the data set. Additional questions were asked about the most important categories of perceived causes and health-seeking. Patient delay was assessed by asking the patients regarding the interval between the onset of symptoms to consultation with any healthcare provider.

Each section of the EMIC began with a short introduction to help the respondents understand the study interests and questions and to encourage them to respond candid and to speak freely. The instrument was prepared in English and translated into the local language (Swahili). Patients’ narrative interview data were translated into English for analysis.

### Variables

#### Outcome variables

Delay in seeking health care was regarded as an outcome variable. Patient delay was defined as a duration of 3 weeks or more between onset of any TB-related symptom (such as coughing, chest pain, fever, weight loss, and hemoptysis) and presentation to any healthcare facility including pharmacies, hospitals, primary healthcare facilities as well as dispensaries [24]. The cutoff point for patient delay of 3 weeks was considered sufficient as described in previous studies [25].

Furthermore, LDFU was regarded as outcome variable. LDFU was defined as failure to present after referral from the recruitment pharmacy to the TB clinic (pharmacy LDFU) within 2 months or failure to return for all three required diagnostic visits after recruitment in the TB clinic (clinic LDFU). Negative and confirmed TB patients who came for timely assessment to all three diagnostic visits were classified as non-LDFU. LDFU was dichotomized as 1 (non-LDFU) or 0 (LDFU).

#### Explanatory variables

Socio-cultural variables specifying features of illness explanatory models included perceived causes that encompass locally held beliefs regarding the common causes of TB. Treatment-seeking referred to the type of healthcare provider patients may consult, including traditional medical practitioners, pharmacies and hospitals [26]. Additionally, sex, age, and socio-economic variables (e.g., education and occupation) were considered as explanatory variables.

A prominence score was assigned to each of the EMIC variables denoting perceived causes and help-seeking options. Prominence was based on whether and how a respondent reported each category. We assigned a value of 2 for a spontaneous response, a value of 1 for a positive response only after probing the category and a value of 0 if the category was still not reported by the respondent when probing. Furthermore, a value of 3 was assigned to those categories that were identified as most important perceived cause or treatment seeking option. This value of 3 was added to the value for how a category was reported (0, 1 or 2). Thus, the values of the prominence score variable had a theoretical range of 0 to 5.

#### Data collection

Clinical officers with research experience administered the interviews after 2 weeks of training. Two clinical officers were present for each interview; one interviewed the patient and the other served as scribe, taking notes. They changed roles in successive interviews. Interviews required 30 to 60 min, and data were collected on paper interview forms.

#### Data analysis

Categorical and numerical variables from the EMIC interviews were double entered using Epi Info (Centre for Disease Control and Prevention, Atlanta GA, USA, version 7). We cleaned the data and performed statistical analysis using Stata version 14.2 (StataCorp, College Station, TX, USA).

We calculated the frequencies of response options and of the derived prominence score values of categories of perceived causes of TB and categories of treatment seeking, analysing mean prominence for the comparison groups.

#### Analysis of perceived causes and health seeking variables

In a separate analysis, we grouped categories representing details of a common theme as in previous studies [27]. For example, perceived cause (PC) response variables for water and food as perceived causes were grouped under ‘ingestion’, a PC group variable. PC response variables for alcohol and smoking were grouped under ‘substance use’. Interview-coded response variables for climate, dust, contamination and airborne illness were all grouped under ‘environmental causes’. Response categories including evil eye/sorcery, demons and the will of god were included in a PC group labeled ‘magico-religious’. A PC group variable labelled ‘contact’ referred to both sexual contact and casual contact with an infected person. A PC group ‘other’ included response categories for heredity, conditions such as fatigue, working too hard and carrying heavy loads (these were explained by some as possibly causing chest pain which later develops into TB). Prominence score values for PC group variables were derived by computing the maximum prominence for each PC response variable in the PC group (0 = not mentioned, 1 = mentioned on probe, 2 = mentioned spontaneously, additional value of 3 if any category in the group had been identified as most important perceived cause). In case of non-linear associations with the outcome the variable was transformed into a categorical variable and included as such in the logistic regression model. To assess the statistical significance of differences between PC response variables, PC group variables and help seeking (HS) response variables for the two sets of comparison groups, i.e., for patient delay and LDFU, we used the Wilcoxon rank sum statistical test.

#### Analysis of factors associated with delay and LDFU

To examine the factors associated with delay and LDFU, we conducted univariate and multivariate logistic regressions. Variables for categories of PC, treatment seeking, and socio-economic factors were summarized with univariate statistics. Multivariate logistic regression models were first run for each subcategory of questions about perceived causes and treatment seeking while adjusting for socio-economic variables. Prominence scores were treated as covariates and linearity of their association with the logit of delay and LDFU was tested by including a quadratic term. Significant non-linearity was only found for ingestion and this variable was then dichotomized into 0 vs. 1–5. Only variables which were associated with the outcome variable with a *p*-value < 0.2 were retained in the final multivariate model except for the variables of perceived causes. We then tested each retained variable for interaction with sex and presented the respective gender-specific estimates.

#### Qualitative data for the integrated analysis

To explain identified differences and associations of the explanatory variables, we analysed narrative data. Narratives from the EMIC interviews were coded and imported in MAXQDA version 12 (VERBI Software, Berlin, Germany). The narratives from EMIC were initially thematically coded with reference to interview questions eliciting the narrative. We used the framework method of Gale et al. [28] for analysis of the qualitative data. The analysis identified emerging themes, patterns, similarities and differences.

Open coding to label concepts were used to define and develop categories, based on properties and dimensions of the respondent’s description [29]. We developed and assigned a list of thematic codes that correspond to each separate section of the EMIC interview (PC, HS, as well as study-specific issues) [23].

## Results

### Sample characteristics

**Fig. 2:**
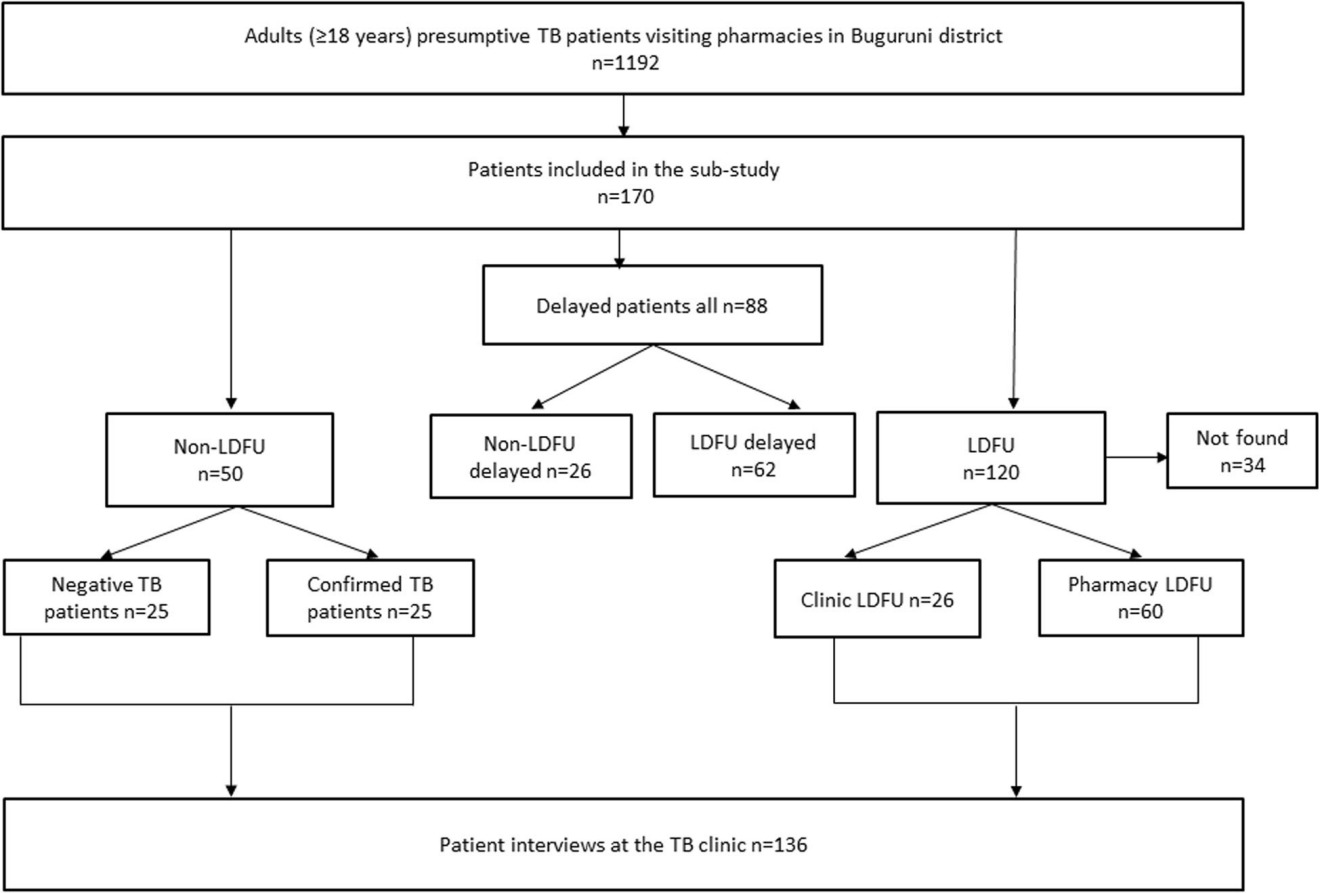
Flowchart of the patient selection (LDFU sub-study)

The study population includes 136 presumptive and confirmed TB patients, of which 86 (63.2%) were LDFU from pharmacies and TB clinic and 50 (36.8%) were non-LDFU (confirmed or negative TB patients). Out of these, patients delay of 3 weeks and more after the onset of symptoms was present in 88 (64.7%) patients, of which 59 (67%) were females (Table 1). Among the 86 patients who were LDFU, 26 (30.2%) did not complete all of the three diagnostic visits at the TB clinic (clinic LDFU). Furthermore, 34 patients in the LDFU group could not be traced and included in the study (Fig. 2). Additionally, in the LDFU group patient delay of 3 weeks and above was present in 62 (72.1%) patients. The median age was 32 years (Interquartile range [IQR]: 27–40 years). Eighty-two (60.3%) of the respondents were females.

**Table 1:** Baseline characteristics of the respondents

Characteristic n (%)	All *n* = 136	Non-LDFU^a^ *n* = 50	Delay^b^ *n* = 88	LDFU^c^ *n* = 86
Age in years, median (IQR)	32 (27–40)	32 (26–36)	32 (26–40)	33 (28–40)
Age group, years				
18–27	35 (25.7)	14 (28.0)	24 (27.2)	21 (24.4)
28–37	57 (42.0)	24 (48.0)	35 (39.8)	33 (38.4)
≥ 38	44 (32.3)	12 (24.0)	29 (33.0)	32 (37.2)
Sex				
Male	54 (39.7)	28 (56.0)	29 (33)	26 (30.2)
Female	82 (60.3)	22 (44.0)	59 (67)	60 (69.8)
Level of education				
Primary/Secondary	118 (86.8)	46 (92.0)	74 (84.1)	72 (83.7)
No formal education	18 (13.2)	4 (8.0)	14 (15.9)	14 (16.3)
Religion				
Muslim	88 (64.7)	32 (64.0)	59 (67)	56 (65.1)
Christian	48 (35.3)	18 (36)	29 (33)	30 (34.9)
Employment status				
Unemployed	36 (26.5)	15 (30)	27 (30.7)	21 (24.4)
Employed	100 (73.5)	35 (70)	61 (69.3)	65 (75.6)
Monthly household income				
Low income	46 (33.3)	17 (34.0)	34 (38.6)	35 (40.7)
Middle income	50 (36.7)	18 (36.0)	29 (32.9)	25 (29.1)
High income	40 (29.4)	15 (30.0)	25 (28.4)	26 (30.2)
Household size				
≤ 4	75 (55.2)	27 (54)	45 (51.1)	48 (55.8)
> 4	61 (44.8)	23 (46)	43 (48.9)	38 (44.2)

*IQR* Interquartile Range, *LDFU* loss to diagnostic follow-up, *Non-LDFU* non- loss to diagnostic follow-up, *1 US $* 2190 Tanzanian Shillings

^a^Including negative and confirmed TB patients who attended the clinic and completed all the visits

^b^Patients who delayed seeking health for ≥3 weeks after onset of symptoms in both LDFU and non-LDFU groups

^c^Including patients who did not visit the clinic after referral from pharmacy and those who did not complete their visits after treatment initiation

Overall, most of the study participants had primary education (86.8%) and were Muslims (64.7%). Patients who had a middle monthly household income were slightly more frequent (36.8%) than those who had low (33.3%) and upper (29.4%) household income.

### Perceived causes

Dust was considered to be a main cause of TB symptoms. 47.7% in the LDFU group and 54.0% in the non-LDFU group spontaneously mentioned dust as the cause of TB symptoms (Table 2).

“I work in the marketplace. My working environment is full of dust; I think this is what caused me to develop TB symptoms” *(male, 41 years).*

**Table 2:** Perceived causes for TB symptoms among LDFU and non-LDFU patients

LDFU *n* = 86					Non-LDFU *n* = 50					
How reported^a^					How reported^a^					
Category^a^	Total reported %	Spont %	Probe %	Most important %	Mean Prominence^b^	Total reported %	Spont %	Probe %	Most important %	Mean Prominence^b^
Ingestion	36.0	5.8	30.2	4.6	0.6	58.0	12.0	50.0	8.0	0.9
Food	24.4	4.7	19.8	3.5	0.4	44.0	6.0	38.0	6.0	0.6
Water	27.9	1.2	26.7	1.2	0.3	32.0	6.0	26.0	2.0	0.4
Substance use	88.3	24.4	63.9	16.3	1.6	84.0	22.0	62.0	10.0	1.3
Alcohol	56.9	5.8	51.2	4.7	0.8	44.0	8.0	36.0	2.0	0.6
Smoking	69.7	20.9	48.8	11.6	1.3	66.0	20.0	46.0	8.0	1.1
Environmental causes	97.7	82.6	15.1	45.4	3.2	98.0	84.0	14.0	44.0	3.1
Climate	74.4	38.4	36.1	6.9	1.3	86.0	46.0	40.0	4.0	1.4
Dust	75.6	47.7	27.9	18.6	1.8	86.0	54.0	32.0	28.0	2.2
Contamination	69.8	27.9	41.8	5.8	1.1	56.0	10.0	46.0	2.0	0.7
Airborne illness	48.8	19.8	29.1	13.9	1.1	40.0	6.0	34.0	10.0	0.8
Magico-religious courses	93.0	70.9	22.0	15.1	2.1	98.0	62.0	36.0	14.0	2.0
Evil eye/sorcery	80.2	45.3	34.9	13.9	1.6	70.0	28.0	42.0	12.0	1.3
Demons or Gods	80.2	45.4	34.9	1.2	1.2	78.0	48.0	30.0	2.0	1.3
Contact	68.6	24.4	44.2	5.8	1.1	62.0	14.0	48.0	8.0	1.0
Casual sexual contact	22.1	5.8	16.3	2.3	0.3	34.0	2.0	32.0	2.0	0.4
Contact with infected person	62.8	20.9	41.9	3.5	0.9	50.0	12.0	38.0	6.0	0.8
Other conditions	68.6	24.4	44.2	10.5	1.2	78.0	24.0	54.0	8.0	1.3
Prior illness	27.9	1.2	26.7	5.8	0.4	38.0	4.0	34.0	4.0	0.5
Heredity	51.2	19.7	31.4	3.5	0.8	60.0	20.0	40.0	4.0	0.9
Others	17.4	3.5	13.9	1.2	0.2	34.0	2.0	32.0	0	0.4

*LDFU* loss to diagnostic follow-up, *Non-LDFU* non- loss to diagnostic follow-up, ^a^ Columns indicate percentage of reported categories and whether a category was identified as most troubling; ^b^ Mean prominence based on values assigned to each reported category (0 = not reported, 1 = reported after probing, 2 = reported spontaneously 3 = identified as most troubling 4 = most troubling and reported after probing 5 = most troubling and reported spontaneously

This was followed by evil eyes and sorcery, 45.3 and 28.0%, respectively. Other common explanations mentioned of TB symptoms were climate, demons or gods.

“My illness might have been caused by the environment at my previous work. As a cook, I was exposed to a lot of smoke and heat. Moreover, my workplace was near a dusty road. Therefore, I was exposed to dust as well. I strongly believe that was the cause of my illness”* (male, 27 years).*

“I work at the market where I often generate more profit than most of my surrounding neighbours. I began coughing blood and became very fragile, I thought my neighbours were jealous of my success and bewitched me. I visited a traditional healer” *(female, 44 years).*

In contrast; alcohol was rarely mentioned spontaneously as a cause of TB symptoms. 51.2, and 36% in LDFU and the non-LDFU group mentioned alcohol as a perceived cause of the disease after probing. This was followed by smoking in both groups (48.8 and 46.0%). Prior illness and contaminated water were reported as the main causes of the disease after probing.

### Treatment seeking

Respondents were asked about their healthcare seeking after onset of symptoms. Seeking help from the pharmacies was considered the most important choice after the onset of symptoms for both females and males in LDFU (Table 3) and non-LDFU groups (Table 4).

“I belived that the pharmacist would understand what is my problem. She gave me some tablets to relieve pain and informed me it would be best to go to the clinic” *(female 57 years).*

**Table 3:** Treatment seeking for TB symptoms among women and men in the LDFU group

LDFU women *n* = 60						LDFU men *n* = 26				
How reported^a^						How reported^a^				
Category^a^	Total reported %	Spont %	Probe %	Most important %	Mean Prominence^b^ %	Total reported%	Spont %	Probe %	Most Important %	Mean Prominence^b^
Home remedies	83.3	45.0	38.3	15.0	1.7	80.7	23.1	57.6	3.8	1.1
Pharmacy	96.7	86.7	10.0	36.7	2.9	92.3	65.4	26.9	38.5	2.7
Hospital	48.3	15.0	33.3	11.7	0.9	46.1	11.5	34.6	3.9	0.6
Traditional healer	76.6	53.3	23.3	21.7	1.9	76.9	42.3	34.6	23.1	1.9
Religious healer	40.0	11.7	28.3	6.7	0.7	42.3	11.5	30.8	11.5	0.8
Dispensary	33.3	8.3	25.0	0	0.4	34.6	23.1	11.5	7.7	0.6
TB clinic	10.0	6.7	3.3	6.7	0.4	23.1	3.8	19.2	3.8	0.3

*LDFU* loss to diagnostic follow-up; ^a^ Columns indicate percentage of reported categories and whether a category was identified as most troubling; ^b^ Mean prominence based on values assigned to each reported category (0 = not reported, 1 = reported after probing, 2 = reported spontaneously 3 = identified as most troubling 4 = most troubling and reported after probing 5 = most troubling and reported spontaneously

**Table 4:** Treatment seeking for TB symptoms among women and men in Non-LDFU group

Non-LDFU women *n* = 22						Non-LDFU men *n* = 28				
How reported^a^						How reported^a^				
Category^a^	Total reported %	Spont %	Probe %	Most important %	Mean Prominence^b^	Total reported%	Spont %	Probe %	Most Important %	Mean Prominence^b^
Home remedies	86.3	54.5	31.8	9.1	1.6	75.0	32.1	43.0	14.3	1.5
Pharmacy	81.8	40.9	40.9	27.3	2.0	96.4	75.0	21.4	28.6	2.6
Hospital	68.2	36.4	31.8	4.6	1.2	71.4	50.0	21.4	7.1	1.4
Traditional healer	81.8	36.4	45.5	22.7	1.7	67.9	39.3	28.6	32.1	2.0
Religious healer	59.1	13.6	45.5	9.1	1.0	39.3	3.6	35.7	3.6	0.5
Dispensary	40.9	18.1	22.7	0	0.5	42.8	7.1	35.7	3.6	0.6
TB clinic	22.8	4.6	18.2	9.1	0.5	21.4	3.6	18.0	0	0.3

*Non-LDFU* non- loss to diagnostic follow-up, ^a^ Columns indicate percentage of reported categories and whether a category was identified as most troubling; ^b^ Mean prominence based on values assigned to each reported category (0 = not reported, 1 = reported after probing, 2 = reported spontaneously 3 = identified as most troubling 4 = most troubling and reported after probing 5 = most troubling and reported spontaneously

Furthermore, among help-seeking options, use of traditional healers was mentioned in both groups, including 21.7% of women and 23.1% of men in the LDFU group and 22.7% women and 32.1% men in the non-LDFU group. Seeking help from religious healers was reported as most important choice among men in the LDFU group compared to women (11.5% vs 6.7%) while in non-LDFU group women reported help-seeking from religious leaders more often as the most important choice compared to men (9.1% vs 3.6%). Furthermore, the majority of those who used religious healers in both groups were Muslims. Narrative elaboration suggests psychological comfort was valued from visiting a traditional healer, especially among women. Other health-seeking practices, such as seeking care at dispensaries and TB clinics, were mentioned less often by both groups of respondents.

“I feel safer to visit a traditional healer. I will rather go to a traditional healer than to the hospital. Doctors at the hospital sometimes mismanage patients” *(female, 27 years).*

### Patterns of distress

Coughing was reported by 89.5% vs 88% of patients of the LDFU and non-LDFU as a major distress associated with the experience of having TB, followed by fever 73% vs 58% respectively. Respondents in both groups rarely mentioned other symptoms such as weight loss, breathlessness, and chest pain.

The narratives suggested that coughing blood (hemoptysis) was a pattern of distress that led to eventually seeking help at the pharmacies and other healthcare facilities.

“I had a low fever and mild cough for a week. I purchased some antibiotics at the pharmacy. Yet I did not feel better and later I started noticing blood in my sputum. I thought it was serious and decided to visit the hospital for more check-ups” *(male, 40 years).*

Additionally, coughing blood was associated with socially enacted stigma. Several narratives indicated the manifestation of stigma to the presumptive TB cases as a result of their symptoms.

“My colleagues were frightened with my condition which included fever and coughing blood. One day they explicitly mentioned that they no longer wanted to stay with me because I was coughing blood” (female, 51 years).

### Factors associated with delay in seeking healthcare

We examined which categories of PC and HS were associated with delay in seeking care after adjusting for socio-demographic and socio-economic variables. In univariate analysis, diagnostic delay of ≥3 weeks was associated with female sex (OR 2.21 95% 1.07–4.54) and seeking help at the traditional healer (OR 2.46 95% 1.10–5.50) (Table 5). In the multivariate analysis, diagnostic delay of ≥3 weeks was positively associated with a mid-range household monthly income (compared to low income) (aoR 3.35 95% CI 1.21–9.21), unemployment (aoR 2.81 95% CI 1.00–7.90), and seeking help from a traditional healer (aOR 2.66 95% CI 1.03–6.84) (Table 6). Additionally, the odds of patient delay for females were higher than for males, (aoR 2.16 95% CI 0.96–4.84). We also observed a positive association with perceived substance use as a cause of TB, which was however not statistically significant. (aoR 1.41 95% CI 0.96–2.06). The association with perceived cause ingestion was not linear, with a significantly reduced risk of delay among patients with prominence scores between 1 and 5 compared to those with a score of 0 (aOR = 0.41; 95% CI: 0.18–0.93). This is underscored by the following quotes:

“I described my symptoms to my colleague. His advice was to consult a traditional healer. The traditional healer prescribed some medications to use for a month and re-visit him after completion. My condition worsened after using the traditional medicines for more than two weeks. I decided to visit the pharmacy” (*female, 35 years).*

**Table 5:** Univariate analysis of factors associated with patient delay for presumptive and TB patients, *n* = 136

Variable	OR	95% CI	*P*-value
Socio-demographics			
Sex			0.03
Male	1 (Ref)		
Female	2.21	1.07–4.54	
Age groups			
18–27	1 (Ref)		
28–37	0.72	0.29–1.77	0.48
≥ 38	0.88	0.34–2.28	0.80
Level of education			0.22
No formal education	1 (Ref)		
Primary/secondary	0.48	0.14–1.55	
Monthly household income			
Low income	1 (Ref)		
Middle income	2.18	0.92–5.17	0.07
High income	1.28	0.53–3.04	0.57
Perceived causes			
Ingestion	0.75	0.53–1.07	0.12
Magico-religious	1.15	0.84–1.56	0.35
Environmental causes	0.94	0.76–1.17	0.61
Substance Use	1.21	0.90–1.65	0.20
Person to person contact	0.97	0.71–1.34	0.89
Other conditions	1.0	0.73–1.35	1.0
Help-seeking			
Traditional healers	2.46	1.10–5.50	0.02
Religious healer	1.27	0.62–2.59	0.50
Hospital	0.85	0.42–1.74	0.67
Home remedies	1.28	0.52–3.12	0.58
Dispensaries	1.09	0.52–2.27	0.81

*OR* Odds ratio, *95% CI* 95% Confidence Interval

**Table 6:** Multivariate analysis of factors associated with delay after onset of symptoms for presumptive and confirmed TB patients, *n* = 136

Variable	aOR	*P*-value	95% CI
Sex			
Male	1 (Ref)		
Female	2.16	0.06	0.96–4.84
Monthly household income			
Low income	1 (Ref)		
Middle Income	3.35	0.01	1.21–9.21
High Income	2.18	0.14	0.77–6.17
Occupation			
Employed	1 (Ref)		
Unemployed	2.81	0.05	1.00–7.90
Perceived causes			
Ingestion			
0	1 (Ref)		
1–5	0.41	0.03	0.18-0.93
Magico-religious	1.11	0.56	0.76–1.63
Environmental causes	1.09	0.57	0.79–1.50
Substance use	1.41	0.07	0.96–2.06
Person to person contact	0.93	0.73	0.64–1.36
Other conditions	1.01	0.94	0.70–1.45
Help-seeking			
Traditional healers	2.66	0.04	1.03–6.84
Religious healer	1.67	0.23	0.71–3.87

CI = 95% Confidence Interval; Delay was defined as three weeks or more after onset of symptoms up to the visit of the TB clinic, OR, Odds ratio. Odds ratio associated with variables of perceived causes refer to a unit increment in the respective variable except for ingestion which showed a non-linear association with delay and was therefore categorized. All the variables listed were included simultaneously in the model

Financial hardship was also mentioned as a reason for diagnostic delay and seeking help at the traditional healers.

“I am from a less privileged family. I could not afford the treatment expenses at the hospital. Traditional healers are cheaper compared to hospitals and other health facilities” *(male, 29 years).*

“I stayed for two months before going to the pharmacy. I just used grinded ginger for my symptoms. The reason was I did not have enough money. If I had enoughn money I would have gone earlier to the pharmacy” *(female 26 years).*

Another factor mentioned by the respondents for the diagnostic delay was substance use example smoking.

“I used to smoke a lot. I stopped because my wife did not like the behaviour. When I started coughing, I thought it was the aftermath of my behavior. I thought since I stopped, the cough will also disappear however it did not. My condition worsened then I decided to visit the traditional healer” *(male 27 years).*

Another factor commonly mentioned by the respondents for the diagnostic delay was the perception of coughing as a ‘normal’ cough. A female respondent said:

“My experience of coughing frequently made me feel it was a normal cough that will eventually disappear with some antibiotics. I took antibiotics for almost a month nevertheless my condition worsened. I visited the pharmacy” *(female, 39 years).*

### Factors associated with LDFU

In univariate analysis, female sex was significantly associated with LDFU (OR 3.51 95% CI: 1.70–7.23) as well as perceived cause ingestion (OR 0.68 95% CI 0.47, 0.98) (Table 7). Furthermore, in multivariate analysis, women were significantly more likely to be lost to diagnostic follow-up (aoR 4.12, 95% CI: 1.78–9.50). The perceived cause “other conditions”, including prior illness, was positively associated also with LDFU (aoR 1.39 95% CI 0.96–2.02) (Table 8). The association between LDFU and perceived cause ingestion was also non-linear, with a significantly reduced risk of LDFU among patients with prominence scores between 1 and 5 compared to those with a score of 0 (aOR = 0.31; 95%.CI: 0.14, 0.70). When stratifying analyses by gender, all odds ratios were larger among women but none of the differences between the gender-specific estimates was statistically significant (Table 9).

**Table 7:** Univariate analysis of factors associated with LDFU for presumptive and TB patients, *n* = 136

Variable	OR	95% CI	*P*-value
Socio-demographics			
Sex			
Male	1 (Ref)		
Female	3.51	1.70–7.23	0.001
Age groups			
18–27	1 (Ref)		
28–37	1.07	0.46–2.51	0.86
≥ 38	1.62	0.65–4.05	0.29
Level of education			
No formal education	1 (Ref)		
Primary/secondary	0.68	0.23–1.93	0.47
Monthly household income			
Low income	1 (Ref)		
Middle income	1.24	0.54–2.86	0.60
High income	0.58	0.24–1.37	0.2
Perceived causes			
Ingestion	0.68	0.47, 0.98	0.04
Magico-religious	1.26	0.93–1.72	0.12
Environmental causes	0.92	0.75–1.14	0.49
Substance use	1.08	0.82–1.43	0.12
Person to person contact	1.09	0.80–1.50	0.56
Other conditions	1.15	0.84–1.56	0.36
Help-seeking			
Traditional healers	1.74	0.79–3.84	0.16
Religious healer	0.55	0.27–1.11	0.09
Hospitals	0.48	0.24–0.98	0.04
Home remedies	1.71	0.71–4.09	0.22
Dispensaries	0.64	0.31–1.30	0.21

*OR* Odds ratio, *95% CI* 95% Confidence Interval

**Table 8:** Multivariate analysis of factors associated with LDFU for presumptive and TB patients, *n* = 136

Variable	aOR	*P*-value	95% CI
Sex			
Male	1 (Ref)		
Female	4.12	0.001	1.78–9.50
Perceived causes			
Ingestion			
0	1 (Ref)		
1–5	0.31	0.005	0.14, 0.70
Magico-religious	1.29	0.18	0.88–1.91
Environmental causes	1.06	0.68	0.78–1.43
Substance use	1.26	0.19	0.88–1.80
Person to person contact	1.13	0.51	0.77–1.66
Other conditions	1.39	0.08	0.96–2.02
Help-seeking			
Traditional healers	1.39	0.48	0.54–3.59
Religious healer	0.51	0.11	0.22–1.17
Hospital	0.54	0.15	0.23–1.24

*CI* 95% Confidence Interval, *OR* Odds ratio, *LDFU* loss to diagnostic follow-up. Odds ratio associated with variables of perceived causes refer to a unit increment in the respective variable except for ingestion, which showed a non-linear association with LDFU and was therefore categorized. All the variables listed were included simultaneously in the model

**Table 9:** Multivariate analysis of factors associated with LDFU for presumptive and TB patients with gender specific estimates, *n* = 136

Variable	Females aOR (95% CI)	Males aOR (95% CI)	*P*-Value (Interaction)
Perceived causes			
Ingestion	0.50	0.16	0.54
Environmental causes	1.17	0.84	0.24
Magico-religious	1.96	0.97	0.14
Substance use	1.48	1.02	0.29
Person to person contact	1.46	0.76	0.99
Other conditions	2.14	0.80	0.14
Help-seeking			
Traditional healers	2.55	0.63	0.40
Religious healer	0.84	0.14	0.14
Hospital	1.47	0.14	0.11

*OR* Odds ratio, *95% CI* 95% Confidence Interval, *LDFU* loss to diagnostic follow-up; Gender-specific odds ratios were obtained from model including gender-specific terms for the respective variable in addition to the other variables listed in the table

Variations in the profile of men and women on reason for LDFU were apparent in the narratives. While women were more concerned with the financial burden to their spouses and family members, men were worried about financial burden resulting from missing work.

“The pharmacist advised me to seek further help for my symptoms at the hospital. I decided not to. I did not want to impose more financial burden to my husband and family as a result of my condition” *(female, 44 years).*

“I was not concerned with the costs of treatment at the hospital. I was more worried that my boss will not understand me if I ask permission to visit the hospital” *(male 41 years).*

Fear of being diagnosed with TB in relation to HIV was recurring from the narratives as well as fear of isolation if diagnosed with TB, although it was not statistically significant in the quantitative analysis.

“The pharmacist gave me a small card to come for further check-ups. I was afraid to be asked to test for HIV. I was not ready to know my HIV status*” (male 25 years).*

“My friend was diagnosed with TB and people were afraid to even eat with him. I thought it would be the same with me that’s why I did not visit the hospital as advised at the pharmacy” *(female 21 years).*

## Discussion

Results from this study indicate that patient delay of ≥3 weeks after the onset of symptoms was observed in almost two third, of the respondents. LDFU from the pharmacies to the TB clinic was observed in 44.1% of the presumptive patients, and a third of the patients who did reach the centre, did not complete all required visits. Pharmacies and traditional healers were main healthcare facilities for treatment seeking after the onset of symptoms.

Seeking care at healthcare facilities with absence or sub-optimal TB diagnostics before reaching TB centers was associated with patient delay. This finding indicates that presumptive TB patients follow a complex pathway to care before reaching suitable healthcare facilities for TB diagnosis and treatment. Undoubtedly, this suggests that a substantial number of presumptive TB patients delay seeking appropriate care as a result of visiting non-professional healthcare providers before TB diagnosis [10, 30, 31]. Consequently, this leads to delay in seeking healthcare after the onset of symptoms.

Women reported being comfortable with the care provided by the traditional healers and that healthcare workers would sometimes mistreat patients in the hospitals. This might indicate abuse and low quality of care in public health facilities in addition to a client’s need for psychological comfort. This finding is in line with research documenting that women reach clinical treatment services through a circuitous route and prefer seeking healthcare from traditional healers and private practitioners [32, 33] instead of directly seeking care at healthcare facilities.

Presumptive patients with middle monthly household income delayed more compared to those with low and high incomes. Possibly, presumptive patients who fell in this group had a higher preference for visiting non-professional healthcare professionals than those with upper and low monthly household income. Furthermore, we believe that these patients used self-medication more frequently after the onset of symptoms which is among determinants of delay as reported elsewhere [34]. Moreover, financial limitations resulting from unemployment were related to patient delay. The economic burden associated with treatment seeking is high for the poor population, which is most at risk of acquiring TB, which has already been noted in other settings [35–37]. This highlights the need for poverty reduction in marginalized communities that will consequently contribute to the reduction of TB in low-resource settings especially in sub-Saharan countries [38, 39].

Furthermore, non-reporting of food or water as potential cause of TB was associated with an increased risk of patient delay. This finding may indicate a tendency of presumptive and TB patients to attribute their TB symptoms to other causes than food and water which in turn lead to early health-care seeking. Our findings contradict findings from a recent study in India that found that attribution of TB symptoms to factors such as water, pollution and cold favoured diagnostic delay in the TB patients [40].

Our results show that seeking help at the traditional healers was associated with delay. One of the factors associated with seeking care at traditional healers was financial constraint. Patients claimed that traditional healers are cheaper than other healthcare provider’s example hospitals. This underscores the need to improve health care services by reducing the costs for diagnosis and treatment while considering the marginalized population. Our results are in line with findings from studies in Tanzania that documented patients consulting traditional healers due to financial constraints before reaching other healthcare facilities [14, 41].

We observed a higher risk of delay among those who prioritized substance use, such as smoking and alcohol, as a cause of TB. These patients may have attributed their TB symptoms to coughing as a result of smoking and alcohol consumption and therefore delayed seeking help in proper heath care facilities. Educating patients on the risk factors for TB could potentially decrease patient as well as diagnostic delay while seeking health care.

LDFU in our study was massive. 44% of the respondents did not show up in the diagnostic centre after referral from the pharmacies and every third patient who did reach the centre, did not complete all required visits. Dropouts at the TB clinic did not differ from patients who did not attend any visit at the clinic at all. Although we could not trace whether these patients had completed TB treatment in other areas, we believe this to be partly the case as supported by other studies which documented tendencies of TB patients to continue with TB treatment elsewhere [42–44].

The odds of LDFU among women were higher than those of males. Absence of financial autonomy made women miss diagnostic follow-up in healthcare facilities as indicated in the narratives. Furthermore women might have simultaneously used traditional methods and medical services instead of directly visiting healthcare facilities for proper diagnosis and treatment [33]. The association between female sex and LDFU in our study is contrary to other studies on adherence and loss to follow-up that found men to be more likely to be lost to follow-up [45, 46].

The category of perceived causes “other conditions”, such as prior illness and heredity, showed a marginally significant positive association with LDFU. Although this finding was not well supported by the narratives, possible explanations for such results might be that patients with prior illness and who were previously treated for TB might have had unpleasant memories [47] and did not want to make the same experiences again. This finding highlights the need for educating patients with regards to the importance of adherence and treatment completion so as to reduce loss to follow-up during treatment.

We found that dust exposures followed by sorcery were considered to be the main causes of TB symptoms in LDFU and non-LDFU. Alcohol and smoking were acknowledged as causes of TB after probing. Since ideas and perceptions about TB influence health-seeking behavior, the mentioned perceived causes influenced delay and diagnostic loss to follow-up. Clearly, this highlights the need for clarification on the perceived causes in the community that might help in shortening delay and LDFU and influencing the right help-seeking behaviour.

Findings from the narratives indicate that respondents often started seeking healthcare after coughing up blood (hemoptysis). Additionally, the absence of blood in sputum was interpreted as a ‘normal’ cough which would not be associated with TB. This suggests that there was a tendency among presumptive and TB patients to minimize the importance of their health conditions and discount the need for further treatment until they experienced some specific symptoms like hemoptysis, subsequently leading to patient delay and LDFU. Our findings are in line with findings from the study on health seeking behaviour among people with cough in Tanzania that showed patients with additional TB symptoms sought care earlier than those with no additional symptoms [4].

Psychosocial distresses such as fear of being diagnosed with TB or HIV were recurring themes in the narratives. TB patients often experience considerable psychological and emotional distress as a result of their condition [23, 24, 48], and presumptive TB patients anticipate the emotional burden of TB and of stigma eventually after being diagnosed with HIV that might lead to delay in seeking health care. Thus, this aspect requires some attention in case finding efforts.

Our study is the first to look at patient delay and loss to diagnostic follow-up among presumptive and confirmed TB patients within an intervention study in Tanzania. However, this study has some limitations. We included a small fraction of the presumptive and confirmed TB cases that participated in the big TB-PHARM intervention study to explore the factors associated with delay and LDFU in-depth. Due to the small sample, our analyses have provided few statistically significant results, and our confidence intervals were wide for some odds ratios However, the quantitative and qualitative findings of the study seemed to be coherent.

Furthermore, we relied on patient’s recall of the duration of the delay in treatment seeking that might have introduced inaccuracy. However, our interviews were conducted with well-trained clinical workers who spent considerable time with the patients to obtain as accurate answers as possible. We attempted to limit inaccuracies by linking questions about delay with memorable events such as onset of symptoms until the first care seeking to a healthcare facility.

Finally, we cannot exclude that there may be further factors that could lead to patient delay or LDFU which were not assessed in this study.

## Conclusions

In conclusion, this study highlights that delay after the onset of symptoms and LDFU after referral from the pharmacies is substantial, especially for women. It also confirms the high proportion of patients consulting traditional practitioners and that this behavior is related to delay but not LDFU. This underscores the need for interventions to ensure that more attention is paid to women’s health needs for timely and sustained TB treatment and improved public awareness to reduce misperceptions of the cause and value for medical treatment. These findings also highlight the need to train and educate traditional healers on TB symptoms so they refer patients to the healthcare facilities for further treatment. The preference of the presumptive and confirmed TB patients to visit sub-optimal and unequipped healthcare facilities for treatment of TB-related symptoms as traditional healers may lead to diagnostic delay and LDFU as indicated in our study. These findings underscore the importance of strengthening the health sector to ensure that these patients are traced and linked to the healthcare facilities assuring early detection and effective treatment.

## Abbreviations

EMIC: Explanatory model interview catalogue
HIV: Human immune deficiency virus
IHI: Ifakara Health Institute
IQR: Interquartile range
LDFU: Loss to diagnostic follow-up
LTFU: Loss to follow-up
NIMR: National Institute for Medical Research
OR: Odds ratio
TB: Tuberculosis
USD: United States Dollar
